# Unveiling the hidden players: exploring the role of gut mycobiome in cancer development and treatment dynamics

**DOI:** 10.1080/19490976.2024.2328868

**Published:** 2024-03-14

**Authors:** Lingxi Li, Xiaowen Huang, Haoyan Chen

**Affiliations:** State Key Laboratory of Systems Medicine for Cancer, Key Laboratory of Gastroenterology and Hepatology, Ministry of Health, Division of Gastroenterology and Hepatology, Renji Hospital, School of Medicine, Shanghai Jiao Tong University, Shanghai Institute of Digestive Disease, Shanghai Cancer Institute, Shanghai, China

**Keywords:** Gut mycobiome, cancer development, cancer treatment, Candida, Malassezia, Aspergillus

## Abstract

The role of gut fungal species in tumor-related processes remains largely unexplored, with most studies still focusing on fungal infections. This review examines the accumulating evidence suggesting the involvement of commensal and pathogenic fungi in cancer biological process, including oncogenesis, progression, and treatment response. Mechanisms explored include fungal influence on host immunity, secretion of bioactive toxins/metabolites, interaction with bacterial commensals, and migration to other tissues in certain types of cancers. Attempts to utilize fungal molecular signatures for cancer diagnosis and fungal-derived products for treatment are discussed. A few studies highlight fungi’s impact on the responsiveness and sensitivity to chemotherapy, radiotherapy, immunotherapy, and fecal microbiota transplant. Given the limited understanding and techniques in fungal research, the studies on gut fungi are still facing great challenges, despite having great potentials.

## 1. Introduction

While the gut microbiome comprises bacteria, fungi, and viruses, research on diseases has predominantly centered on understanding the interaction between the host and gut bacteria. This emphasis arises from the fact that the intestinal bacteriome constitutes more than 99.9% of the overall gut microbiome.^1^ Fungi, constituting the remaining 0.1%, however, also play a crucial role in normal and pathological mechanisms, akin to bacteria. Fungi establish early colonization in the intestine during birth, breastfeeding, or through ingestion via food, respiratory tracts, or skin-mouth contact. Food is another significant fungal source. The gut mycobiota is mainly composed of *Ascomycota*, *Basidiomycota*, *Chytridiomycota*, and *Zygomycota*. Prominent commensal fungi species in the human gut include *Candida spp*., *Saccharomyces spp*., and *Malassezia spp*.^2^ Normally nonpathogenic, these fungi can become serious threats when the host’s homeostasis is disrupted, particularly in cases of a suppressed immune system or reduced bacterial population. Fungi exhibit morphological adaptability, capable of transitioning between unicellular and multicellular forms, particularly yeast and hyphae, respectively, which influences their pathogenicity. Hyphae typically exhibit higher invasiveness, while yeasts are usually non-intrusive and often linked to mutualistic relationships.^3–5^ Fungi also demonstrate the ability to generate both harmful and beneficial compounds. Utilizing toxic metabolites and proteases, fungi can switch between various morphological states, aiding in adhesion, host barrier penetration, and subversion of the host immune response. Recent studies affirm that specific gut bacteria possess the ability to trigger tumorigenesis in genetically susceptible murine models.

Certain types of tumor, especially colorectal cancer (CRC), have been extensively studied, with emphasis given to *Enterotoxigenic Bacteroides Fragilis*, *Escherichia coli*, and *Fusobacterium nucleatum* as key components in their pathogenesis.^6^ Limited research has focused on the tumorigenesis of intestinal fungi. Nevertheless, existing studies suggest a potential connection between diverse cancer types and various fungal species.

## 2. Common pathogenic mechanisms

**Figure 1.**
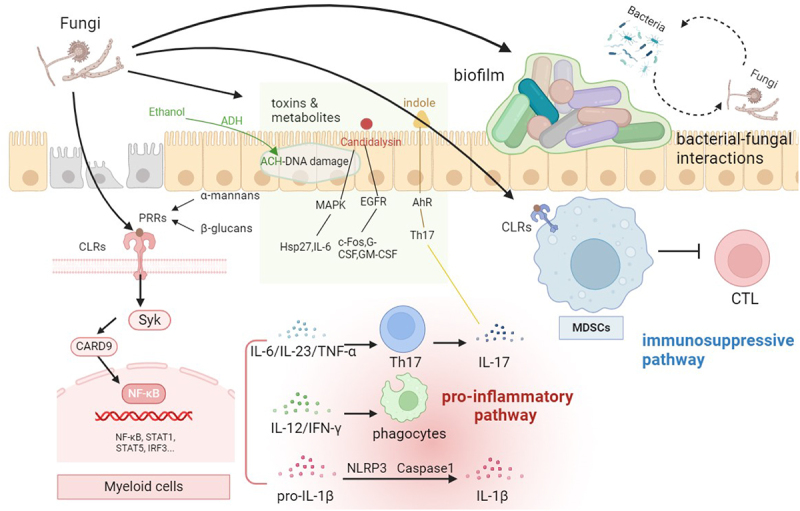
The common mechanisms of fungi that are involved in triggering pathogenesis, especially in oncogenesis. PRRs, pattern recognition receptors; CLRs, C-type lectin receptors; CARD9, Caspase Recruitment Domain-containing protein9; STAT1, signal transducer and activator of transcription 1; Th17, T helper 17 cell; IL-6, interleukin 6; IL-23, interleukin 23; TNF-α, tumor necrosis factor α; IL-12, interleukin 12; IFN-γ, interferon γ; IL-1β, interleukin 1β; MDSCs, myeloid-derived suppressor cells; TCL, cytotoxic T cell; ACH, acetaldehyde; MAPK, mitogen-activated protein kinases; EGFR, epithelial growth factor receptor; AhR, aryl hydrocarbon receptor; Hsp27, heat shock protein 27; G-CSF, granulocyte colony-stimulating factor; GM-CSF, granulocyte-macrophage colony-stimulating factor.

Over the decades, studies have been majorly focused on the infections and septicemia caused by gut fungi. However, there are still studies implicating the possible common mechanisms by which gut fungi are able to induce host immune and microbiome dysregulation, and subsequently leading to oncogenesis. These may include the fungi–host recognition and immune regulation, biofilm formation, bacterial–fungal interactions, and toxins and metabolites produced by them (Figure 1).

### 2.1. Fungi-host recognition and inflammatory signaling pathway

C-type lectin receptors (CLRs), typically Dectin-1, Dectin-2, Dectin-3, and Mincle, are subtypes of pattern recognition receptors (PRRs), primarily expressed on myeloid cell surfaces including surfaces of macrophages, neutrophils, and dendritic cells. These CLRs detect fungal cell wall components like β-glucans and α-mannans, engage in sensing fungal infections and activating downstream signaling pathways, triggering inflammation and immune responses.^7^ This ligand-receptor binding activates multiple intracellular signaling pathways, including adaptor molecules, kinases, and transcription factors. The Caspase Recruitment Domain-containing protein9 (CARD9) is an essential adaptor protein restrictedly expressed in myeloid cells and is critical in initiating inflammatory caspase in response to *Candida*, *Malassezia*, and *Aspergillus*. CLR requires CARD9 for subsequent innate immunity activation.^8–11^

Dectin-1 signaling triggers the segmentation of pro-IL-1β, resulting in the ultimate production of IL-1β through activation of NLRP3 and caspase-1 or caspase-8 inflammasome pathways.^12–14^ The NLRP3 inflammasome is potentiated by diverse activators and recognizing patterns, including damage-associated molecular patterns (DAMP), and pathogen-associated molecular patterns (PAMP), which are targets of fungi, bacteria, and viruses.^15^ Subsequently, downstream immune response is triggered by these activators in the different circumstances, including fungal invasions and infections.

NLRP3 inflammasome activation is found to be associated with two stepwise signals. Signal 1 initiates the immune recognition response such as recognition by Dectin-1 mentioned above, followed by activation of nuclear factor-κB (NF-κB), driving the production of pro-IL-1β. The signal 2 involves subsequent activation of caspase-1, resulting in the maturation of IL-1β.^16,17^

Dectin-3, also known as CLED4D or CLECSF8, operates as a PRR in various myeloid cells. It recognizes multiple fungi, including *C. albicans* and *Cryptococcus*, by detecting α-mannans on their surfaces. A prior study conducted by Zhu et al. revealed that Dectin-3 forms a heterodimeric complex with Dectin-2 to detect α-mannans.^18^

Recognition of fungi such as *C. albicans* activates pathways like MAPK, STAT1, STAT3, NF-κB, and IRF3, leading to the production of mediators such as interleukins (IL-1, IL-6, IL-12, and IL-23), tumor necrosis factor-α (TNF-α), and interferon gamma (IFN-γ).^19–22^ These responses contribute to both innate and adaptive immunity.^23^ The inflammatory activation pathways and interferons further promote the regulation and alteration of the host immune response, playing a crucial role in the development of both malignant and benign conditions. *Malassezia* can elicit cooperative recognition by Mincle and Dectin-2 through distinct ligands that cause skin inflammation.^24^

The CLRs, accompanied by the subsequent downstream activation of variable signaling pathways, actively participate in the regulation of immune responses, and thus affecting disease progression.

### 2.2. Immune cell activation

Myeloid-derived suppressor cells (MDSCs), composed of granulocytic MDSCs (G-MDSCs) and monocytic MDSCs (GM-MDSCs), constituting a heterogeneous group of immature myeloid cells. Their role involves promoting immune suppression and aiding in tumor development. Fungal dysbiosis leads to an increase in the release of MDSCs, thus contributing to tumorigenesis. In CRC patients, a positive correlation occurs between the abundance of MDSCs at tumor sites and the fungal burden. Several transcriptional factors, including STAT3 and IRF8, have been implicated in the expansion and differentiation of MDSC.^25–29^

Th17 cells, an antigen-specific subset of CD4^+^ T cells, contribute to coordinating immune responses against extracellular bacteria and fungi. Th17 cells, initially activated by *C. albicans*, broaden their response to target other fungi through cross-reactivity. Intestinal inflammation expands *C. albicans*-specific and cross-reactive Th17 cells.^30^ Dectin-SyK-CARD9 signaling pathways can stimulate the secretion of interferons and cytokines, as well as the differentiation of T lymphocytes into IL-17-producing CD4^+^ T cells.^31,32^ Pro-inflammatory cytokine IL-17 secreted by Th17 cells is regarded as a contributor to multiple autoimmune diseases including autoimmune thyroid disease,^33^ atopic dermatitis,^34^ IBD,^35^ etc. Studies have explored its role in tumor development in mice, revealing that indole’s carcinogenic effect involves interfering the Th17 cell differentiation and IL-17 production under AhR regulation.^36^ Additionally, IL-23, a cytokine crucial for expanding and sustaining Th17 cells, is vital for inducing T-cell mediated colitis in murine models.^37^ Moreover, it has been observed to foster angiogenesis and contribute to tumor growth.^37–39^

MDSCs and Th17 cells significantly promote immune suppression and tumor development. Fungal dysbiosis induces increased MDSC release and Th17 differentiation, contributing to tumor progression. Th17 cells, initially activated by *C. albicans*, amplified their response to target fungi and contributes to inflammation. The stimulation of stimulate Th17 differentiation and IL-17 production is assisted by the Dectin-SyK-CARD9 pathways, as implicated in autoimmune diseases and tumor development.

### 2.3. Bacterial–fungal interactions

Bacteria and fungi can interact synergistically, mutually, or antagonistically, shaping the microbiome’s unique features in different human body parts and health states. Bacterial species are often found accompanied with fungi in the formation of polymicrobial biofilms, such as *Streptococcus mutans*, *P. aeruginosa*, and *S. aureus* .^40^ Infections originating from biofilms that involve both bacteria and fungi pose a significantly greater challenge for treatment compared to those involving a single species. Addressing these infections requires intricate multi-drug treatment approaches.^41^ The host-bacterial-fungal interactions can affect the survival, morphogenesis, and invasiveness of fungi.^42^ Bacteria can inhibit the growth of *C. albicans* through the secretion of molecules, metabolites, and factors that function directly on fungi or indirectly through host response.^43–46^ For example, in murine models, *Bacteroides thetaiotaomicron* upregulates the transcription factor HIF-1α and anti-microbial peptide LL-37, initiating host innate immune response to inhibit *C. albicans* colonization.^44^ Notably, it is also said that *B. thetaiotaomicron*-processed mucins enhance *C. albicans* growth more than unprocessed mucins, and thus shaping its spatial distribution.^47^ Short-chain fatty acids (SCFAs), produced by bacterial metabolization and fermentation of non-digestible dietary fibers, typically *Lactobacillus*, are found to hinder hyphal morphogenesis and promote T cell response to *C. albicans* in mice, and finally inhibit biofilm formation and prevent invasion.^45,48^

Bacteria can exploit the microenvironment established by fungi for their growth. The hypoxic microenvironment generated by *C. albicans* in its biofilm is conducive to the growth of anaerobic bacteria such as *Clostridium perfringens* and *Bacteroides fragilis* .^40^ *B. thetaiotaomicron* is known to possess enzymes specialized in breaking down α-mannan, a typical cell wall component of yeasts.^49^ The growth of anaerobic *Bacteroides* species such as *B. vulgatus* and *B. fragilis* are also benefited from metabolizing fungal components in vitro.^50^ Several bacteria, including *Streptococcal species*, *Fusobacterium nucleatum*, and *Helicobacter pylori*, have been documented to exhibit synergistic relationships with *C. albicans*, amplifying their virulence.^51^

A study revealed that *C. albicans* influence the recolonization of cecal microbiota in mice treated with antibiotics. This resulted in an elevation of *Enterococcus faecalis* and a decrease in probiotic *Lactobacillus* strains.^52^ A subsequent investigation demonstrated that intestinal *C. albicans* in antibiotic-treated mice elicit altered expression of specific genes regarding inflammation and host response, resulting in a shift in bacterial diversity while not inducing gut inflammation, supporting the role of *C. albicans* in helping with the recovery of bacterial community.^53^

The dysbiosis of either of bacteria or fungi can cause the dysbiosis of the other. The administration of antibiotics can induce fungal dysbiosis and the overgrowth of *C. albicans* .^54^ Jiang et al. demonstrated that diverse fungal species can effectively substitute for enteric bacteria in addressing infectious and inflammatory disorders, including colitis resulting from the elimination of commensal bacteria.^55^ Van Tilburg Bernardes et al. illustrated that fungal colonization leads to significant alterations in the ecology of the bacterial microbiome. Furthermore, the co-colonization of bacteria and fungi is associated with an elevated level of colonic inflammation.^56–58^

In all, bacteria and fungi interact in various ways, forming polymicrobial biofilms and influencing each other’s growth and virulence. Bacteria inhibit *C. albicans* through diverse mechanisms, while fungal biofilms can create favorable environments for anaerobic bacteria. Dysbiosis in one can disrupt the other, with fungi potentially substituting for bacteria in addressing inflammatory disorders. This interplay significantly alters microbiome ecology and can impact the course and severity of pathogenic status such as colonic inflammation.

### 2.4. Invasiveness and biofilm formation

Biofilms are closely packed communities of cells that attach to biotic and abiotic surfaces, including the intestine.^59,60^ *C. albicans* biofilms exhibit a highly organized structure, incorporating yeast-forming cells, pseudohyphae, and hyphae enveloped by an extracellular matrix. These biofilms participate in the survival of *C. albicans*. *C. albicans* biofilms are typically formed following three stages: yeast cell adsorption and adherence, hyphae development with microcolony formation and extracellular matrix (ECM) production, and maturation with cell dispersal for potential dissemination to secondary infection sites.^61,62^ The formation process may be influenced by several genes: *TEC1*, *BCR1*, *ALS3*, *HWP1*, and *EFH1* .^63–68^ The ECM is composed of self-produced extracellular biopolymers within the biofilm. Macromolecules, such as protein, lipid, and various polysaccharides including α-mannans and β-1,6-glucans,^69^ as well as plentiful of host cells such as neutrophils exist, indicating host engagement in the formation and maintenance of these biofilms.^70^

Quorum sensing factor farnesol, initially identified in high-density cultures, serves as a crucial inhibitor of hyphal formation and participates in the dispersal of biofilm cells of *C. albicans* .^71^ Farnesol hinders the maturation of dendritic cells, and thus causing failure in inducing proper T cell response and innate immunity against *C. albicans* .^72^ *Candida* biofilms offer enhanced protection against the immune system and antifungal therapy compared to planktonic cells. Factors like hyphal morphology, ECM, farnesol, resistance-associated gene expression, fungal-bacteria interactions, persister cells, and biofilm extracellular vesicles all contribute to this mechanism.^61,73,74^

*C. albicans* possess a strong propensity for invasion through the epithelial cells in their hyphae form, an important factor in their pathogenesis.^75,76^ Enzymes like enolase and phytase possess the ability to anchor to the membrane, contribute to hyphal growth, host fibronectin, and laminin binding, epithelial tissue damage, and nutrient acquisition.^77,78^ Although much have been concerned about *Candida* biofilm and infection, the relationship between fungal biofilm and carcinogenesis has seldomly been reported, except that in oral cancer.^79^ The specific features of biofilm, especially its resistance to host immune system, and strong capacity of invasion and dispersion may play a role in the tumorigenesis process.

Fungal species other than *C. albicans* are also capable of forming biofilms and causing biofilm-related infection. Some *Malassezia* species such as *Malassezia pachydermatis* and *Malassezia furfur* are also found to be able to form biofilms in vitro, and these biofilms also possess the ability of pathogenesis and drug resistance.^80,81^ *Aspergillus* ,^82^ *Cryptococcus*, ^83^ and many other fungi also possess the ability to form biofilms and act differently on the pathological process.

In other words, biofilms are crucial for the survival of fungi, especially *C. albicans*, exhibiting structured layers composed of yeast, pseudohyphae, and hyphae within an extracellular matrix. Factors including farnesol, ECM, and fungal-bacteria interactions enhance *C. albicans* biofilm resistance against immunity and antifungal therapy. The invasiveness of the hyphae form of *C. albicans* contributes to its pathogenesis. While rarely reported, fungal biofilms may impact carcinogenesis, exemplified in oral cancer.

### 2.5. Toxins and metabolites

#### 2.5.1. Candidalysin

Candidalysin is a cytolytic toxin produced by *C. albicans* that enables its capacity to damage and evade through host epithelium, and is encoded by the hypha-specific *ECE1* gene.^84^ Candidalysin activates the MAPK pathway, triggering host immune responses such as heat shock protein (Hsp) activation, IL-6 release, and neutrophil recruitment.^85^ However, the detailed signaling caspase involved is still unknown. A recent study highlighted the significance of EGFR activation in candidalysin-induced MAPK signaling. This activation leads to the induction of the c-Fos transcription factor, ultimately triggering neutrophil recruitment through G-CSF and GM-CSF. The process relies on the release of EGFR ligands and calcium influx.^86^ While S. A. Nikou et al. mentioned another mechanism of candidalysin in oral epithelial cells that involves the activation of EGFR and c-Fos independent of MAPK.^87^ Rogiers et al. revealed the pivotal role of candidalysin in eliciting *C. albicans* to induce NLRP3 inflammasome responses, leading to the maturation and secretion of IL-1β in macrophages.^88^ Additionally, *C. albicans* mutants that are unable to transit from yeast to hyphae exhibited compromised TCRαβ^+^ cell proliferation and diminished IL-17a expression in oral epithelial cells. This suggests a potential role of candidalysin in engaging in the recognition and induction of innate immunity of fungi as mentioned above that involves IL-1β and IL-17.^89^

#### 2.5.2. Acetaldehyde

*C. albicans*, as along with some *Candia* species other than *albicans* such as *C. glabrata* and *C. tropicalis*, are able to produce acetaldehyde (ACH) in vitro.^90^ ACH is a genotoxic compound involved in ethanol metabolism, disrupting DNA repair processes and inducing oxidative stress. Dysbiosis can increase local ACH production by opportunistic pathogens, forming a causal link with oral and gastrointestinal carcinogenesis. High ethanol-derived ACH-producing *Candida* were more prevalent in oral cancer than non-oral cancer patients.^79^

#### 2.5.3. Indole

Indoles are alkalic molecules transformed by tryptophan, including indirubin, indolo (3,2-b) carbazole, and FICZ. They can be produced by *Malassezia* metabolism.^91,92^ Indole compounds are robust ligands and agonists of the aryl hydrogen receptor (AhR), and they are associated with skin carcinogenesis.^93^ Abundant AhR, malassezin, and indoles can be observed in *M. furfur* yeast strains causing skin infections, and is related to disrupted TLR-associated dendritic cell maturation.^92^ Kynurenine, a key oncometabolite in the tryptophan pathway linked to numerous diseases, serves as a ligand activating AhR in cells. Blocking kynurenine has proven to inhibit the growth of CRC cells in colon organoids.^94^

Mycotoxins such as aflatoxin patulin and Xaralenone, which are produced by exogenous fungi are also associated with carcinogenesis, despite largely unexplored mechanisms.^95–97^ *Aspergillus* species are able to produce aflatoxin that is a well-studied carcinogenic mycotoxin for hepatocellular carcinoma (HCC) development, and we will mention it in detail later.

## 3. Specific fungi in oncogenesis

**Figure 2.**
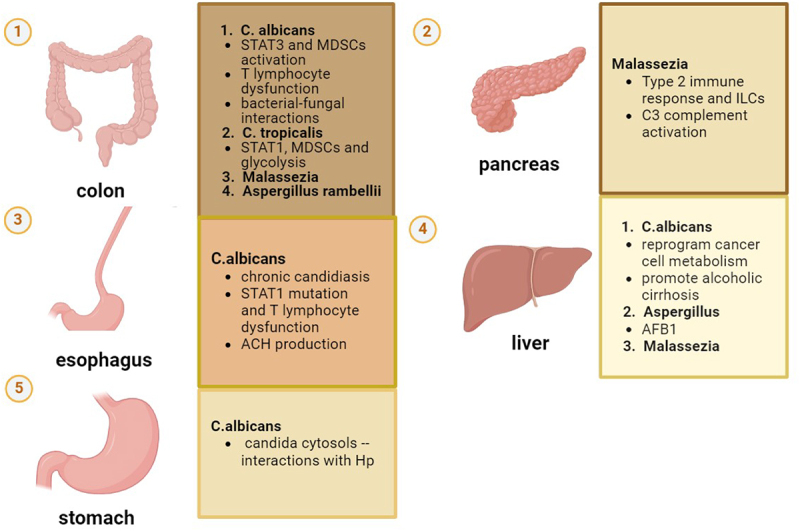
Fungi and their roles in specific tumors. Shown are the various mechanisms through which major gut fungi species are able to influence the specific cancers, including the immune responses, cytotoxins and metabolites, and bacterial–fungal interactions

Similar to bacteria, specific gut fungal genus, and species display an augmentation in the intestine of specific tumor patients, and they may contribute to tumor progression through different mechanisms (Figure 2). They are suspected to be a cause even if the tumor does not originate along the digestive tract, and the possibility of migration helps to explain this phenomenon.

### 3.1. Candida

*Candida* is commonly manifested as a commensal component of the human gastrointestinal tract. However, it can become pathogenic due to various host-related factors such as gut microbiome disruption, immunocompromised status, and significant metabolic changes. While candidemia is the most direct manifestation, *Candida* is also associated with multiple types of cancer. Its role in oral cancer is the most extensively explored,^98^ with reported but inconclusive evidence of its involvement in other cancers including colorectal cancer, neck and cervical cancer.

#### 3.1.1. Candida Albicans

*C. albicans*, in normal health status, presents as harmless native commensal microbiota colonizing mucosa at multiple sites, including mouth, skin, the gastrointestinal tract, vagina, and vascular system.^64,65,68^ However, it also acts as an opportunistic pathogen capable of causing severe and potentially fatal bloodstream infections, particularly in both immunocompromised and immunocompetent individuals. *C. albicans* can hematogenously disseminate, reaching multiple organs and potentially leading to serious complications. *C. albicans* displays significantly increased pseudohyphae morphogenesis, virulence factor secretion, and biofilm formation compared to certain non-albicans Candida species such as *C. auris*, *C. tropicalis*, *C. parapsilosis*, *C. haemulonii*, and *C. glabrata*, observed in vitro studies and in murine models.^99,100^
*C. albicans* can potentially drive cancer progression through multiple mechanisms, such as the generation of carcinogenic byproducts, initiation of chronic inflammation, alteration of the immune microenvironment, activation of oncogenic signaling pathways, and interactions with bacteria. Here, we summarize the possible relationship between cancers and *C. albicans* in or derived from the gut.

##### 3.1.1.1. Esophageal cancer

Esophageal cancer (EC) has been found to be associated with *Candida* infection, of which *C. albicans* are the most relevant.^101^ It is more prevalent in patients with EC than in those with esophagitis (27% compared to 15%), *Candida* infection may be correlated to the development of EC.^102^ On average, patients with fungal infections are 7 years younger than those with EC, suggesting fungi as potential triggers for EC initiation and development.^103^

Chronic esophageal candidiasis is primarily observed in immunocompromised conditions, including chronic mucocutaneous candidiasis (CMC), which is a common clinical symptom of autoimmune polyendocrinopathy-candidiasis-ectodermal dystrophy (APECED). APECED patients demonstrate an impairment of negative selection of autoreactive T cells resulting in autoimmunity, rendering them vulnerable to chronic bacterial and fungal infections and esophageal squamous cell carcinoma (ESCC), as evidenced by multiple case reports.^104–106^ Patients with *C. albicans* may experience differing degrees of T-lymphocyte dysfunction, potentially increasing their susceptibility to esophageal cancer, proven by the fact that fungal depletion and administration, respectively, inhibits and accelerates ESCC development.^107^ Delsing and Koo suggested a relationship between *STAT1* function deficiency, autoreactive CD4^+^ T cells, and chronic fungal infection. Koo et al. documented a family with CMC and a confirmed gain-of-function *STAT1* mutation. The patients, who experienced CMC in childhood, presented severe oral and esophageal candidiasis and were at risk of developing oral cancer and/or ESCC.^106^ Delsing et al. expressed concern about nitrosamine production, and defective T-lymphocyte function against oncogenesis along with the *STAT1* mutation in the development of esophageal carcinoma.^105^ Another potential mechanism could be the local production of ACH by *C. albicans*, given the significant microbial composition similarity between the esophageal mucosa and the oral cavity.^108,109^

##### 3.1.1.2. Gastric cancer

*H. pylori* has long been acknowledged as the most important risk factor for gastric cancer (GC). However, whether other microbials take their part in the development of GC remains largely unexplored.

In 2014, Sano et al. showed that infection caused by orally or intravenously administrated *C. albicans* leads to chronic hyperplastic candidiasis and ultimately induces oncogenic processes in the stomach in diabetic rat models.^110^ Zhong et al. later substantiated the hypothesis by revealing the notable fungal dysbiosis in the intratumor biopsy samples, characterized by a significant increase of *C. albicans* abundance and a minor increase in other species such as *Fusicolla acetilerea* and *Arcopilus aureus* .^111^ This enrichment of *C. albicans* is also shown in a pan-cancer analysis.^112^ Furthermore, a case report mentions the possibility that genetic mutations and *C. albicans* infection corporately induce GC development, both leading to defective production of IL-17.^113^ Intravacuolar *H. pylori*, found within *Candida* cytosols in mice, indicates endosymbiosis function of *C. albicans*. This protective environment within *C. albicans* vacuoles may participate in promoting the virulence and survival of *H. pylori*, potentially contributing to tumor development.^114^

Contrarily, Zhang et al.‘s in silico study found no difference in *Candida* species abundance between tumor lesions and surrounding normal tissues in 61 GC patients. Instead, a significant enrichment in the *Solicoccozyma* was observed.^115^ Furthermore, a prospective cohort study conducted by Ndegwa et al. revealed no associated risk of *Candida*-related oral lesions with the development of GC.^116^

##### 3.1.1.3. Colorectal cancer

Until now, there have been no consistent perspectives on the role of *C. albicans* in CRC development. Patients with inflammatory bowel disease are always regarded as a high-risk population of CRC. Prior studies on inflammatory bowel disease have identified fungal community dysbiosis, manifested with an elevated abundance of intestinal *Candida* species and *Malassezia* species.^117–119^ There are studies showing no increase in *albicans* abundance neither in CRC nor in adenoma patients in their intratumor biopsy samples.^120,121^ Gao et al. even reported the considerable decrease in the number of *Candida* species in CRC.^120^ Furthermore, Jin et al.'s research revealed that, despite the fact that high-fat diet is associated with dysbiosis and alteration of multiple bacterial and fungal species, the abundance of gut *Candida* was reduced other than increased in mice that developed CRC.^122^

Anti-fungi immunity, especially for *C. albicans*, sensed via Dectin-Syk-CARD9 pathway, is found to be a protective factor for colitis-associated cancer (CAC). In *Card9*^−/−^ mice, fungal dysbiosis was observed, leading to increased STAT3 activation-an essential transcription factor for MDSCs and a significant driver for CRC tumorigenesis.^26,123^ In Zhu’s study, the elevated *C. albicans* load in *Dectin-3*^−/−^ mice load triggers glycolysis in macrophages and IL-7 secretion. The IL-22 production followed by IL-7 production in innate lymphoid cells are implemented through the AHR and STAT3 experiments.^124^ Loss of Dectin-3 also leads to decreased inflammasomes and IL-18 activation, leading to impaired CD8^+^ T cell function, decreasing epithelial barrier restitution and IFN-γ production in mice.^125^

Although distinct relationship has not been discovered, bacterial–fungal interactions can be highly suspected in the tumorigenesis process. Sovran et al. proposed a bacterial–fungal interaction affecting DSS-induced colitis in mice. Antibiotic treatment altered susceptibility and the impact of fungi on colitis, with *C. albicans* exacerbating colitis in untreated settings.^57^

##### 3.1.1.4. Liver cancer

Recently, the possible link between liver disease and *C. albicans* has been taken into consideration, and mechanisms explored include translocation of β-glucan,^126^ Th17 cells,^127^ and candidalysin^128^ derived from or secreted by intestinal albicans, as well as the activation of Dectin-1 in Kupffer cells, and subsequent increase in IL-1β expression.^129^ Nevertheless, still little is known about whether gut fungi count in HCC, although some bacterial species are found to be correlated.^130^

The gut mycobiome showed decreased diversity in HCC patients among various studies.^131,132^ Despite this, *C. albicans* has undergone an enrichment in the gut of HCC patients. In tumor-bearing mice, *C. albicans* was observed to promote HCC by reprogramming NLRP6-dependent cancer cell metabolism.^131^ *C. albicans* was found to be significantly more abundant in late stage HCC.^132^ Usami et al. suggested a likelihood between intestinal *Candida* and serum fatty acid metabolism in HCC. They found strong positive correlations (coefficients 0.81 and 0.88) between serum EPA (eicosapentaenoic acid), EPA/AA (arachidonic acid) ratio, and fecal *Candida* in the liver cirrhosis (LC) group of 46 HCC patients.^133^

As a commensal microbe, *C. albicans* exhibits higher virulence compared to other *Candida* species, contributing to cancer progression via carcinogenic byproducts, chronic inflammation, and altered immune microenvironment. In esophageal cancer, *C. albicans* infection correlates with younger onset and autoimmune conditions; gastric cancer may be influenced by fungal dysbiosis and the protective effect of *C. albicans* on *H. pylori*, potentially promoting oncogenesis; in colorectal cancer, the role of *C. albicans* are most studied with the involvement of Dectin-Syk-CARD9 pathway, with bacterial–fungal interactions suspected; liver cancer studies suggest *C. albicans* enrichment in HCC patients, impacting metabolism and disease progression. Also, *C. albicans* colonized in the mucosa other than the gut can influence site-specific tumors with various mechanisms. *C. albicans* in the oral cavity is related to oral pre-cancer lesions and can directly induce oral cancer through IL-17 activation, biofilm formation, oncogenic metabolites, and mycotoxin production, such as candidalysin, nitrosamine, and ACH.^76,134^ Interestingly, oral *albicans* have been found to be correlated with blood malignancy in several cohorts.^135,136^

#### 3.1.2. C. tropicalis

*C. tropicalis* was found to be closely related to several stages in the pathological formation of colon diseases, including ulcerative colitis, formation of adenoma, oncogenesis process of CAC, and resistance to chemotherapy.^121,137–139^ Until now, the majority of the observed mechanical processes are associated with Dectin-Syk-CARD9 pathway. *C. tropicalis* is specifically increased in *Dectin-3*^−/−^ and *Card9*^−/−^ CAC mice, and antifungal treatment ameliorated CAC in the previous study.^26,125^ Deficiency of Dectin-3 impairs phagocytic and fungicidal abilities of macrophages in *C. tropicalis* and thus promotes colitis.^125^ The increase of *C. tropicalis* is demonstrated to enhance the accumulation of MDSCs in *Card9*^−/−^ mice.^26^ However, further research indicated that *C. tropicalis* promotes tumorigenesis through influencing aerobic glycolysis by the Dectin-3-Syk-PKM2-HIF-1α glycolysis signaling axis, which involves MDSCs.^138^ Toxic products such as NADPH oxidase 2 and reactive oxygen species (ROS) are produced and lead to T cell dysregulation.^138^ Their further investigation revealed that the *C. tropicalis* facilitates the NLRP3 inflammasome production through the Dectin-3-glycolysis pathway, and finally leads to the increase in IL-1β production on MDSCs. The wholistic process involves activation of several transcription factors and signaling molecules, including STAT1, pro-caspase-1, mitochondrial ROS, and finally inducing IL-1β production. The elevation of these participants to immune pathways may suggest another target for CRC treatment.^17^ A new potential mechanism is asserted by Qu et al. that *C. tropicalis* can interfere with tumor PD-1 expression by increasing tumor cell autophagy and thus promote CRC progression.^140^

### 3.2. Malassezia

To our recent knowledge, *Malassezia* is composed of 18 species, most of which colonize the skin, but can also colonize other mucosal surfaces such as human gut, as well as abiotic surface that form biofilms.^141^ *Malassezia* genus has been reported to be the second most abundant genus among all human stools in proportions from 2 to 4%, while *M. restricta* (more than 80%) and *M. globosa* (36%) account for the most prevalent species.^142,143^

#### 3.2.1. CRC and liver cancer

While *Malassezia* has been extensively studied as an important contributor to the tumorigenesis of skin and breast cancer due to its skin colonization,^144^ there is relatively limited research on its impact on cancers resulting from gut colonization.

An increased abundance of *Malassezia spp*. and fungal dysbiosis was found in IBD patients,^117^ as well as in CRC patients.^145,146^ Interestingly, one study also mentioned an increased abundance of *Malassezia* species in HCC patients compared with cirrhosis and healthy controls.^132^

Unfortunately, few studies have been dedicated to the underlying mechanisms of gut *Malassezia* in gastrointestinal oncogenesis so far, and mountainous research needs to be done to clarify the exact relationship. A possible mechanism may lie in the metabolism of tryptophan into indole that is associated with kynurenine production.^94^

#### 3.2.2. Pancreatic cancer

While there is extensive discussion on the role of bacteria, such as *Fusobacterium sp*., *Enterobacteriaceae*, and *H. pylori*, in pancreatic cancer tumorigenesis, limited efforts have been paid on the involvement of fungi in this oncogenic process.^147^ Research on tumorigenesis in KC mice (*p48*^*cre*^;*LSL-Kras*^*G12D*^), a model expressing oncogenic *Kras* in pancreatic progenitor cells for slow progressive pancreatic ductal adenocarcinoma (PDA), demonstrated significant fungal dysbiosis in the pancreas, with *Malassezia spp*. being the predominant genus.^148^ Unlike the gut or normal pancreas, both mouse and human PDA mycobiomes featured increased *Malassezia* species. Notably, *Malassezia* in the pancreas tissue is shown to have migrated from the gut.^148^

Dectin-1 is not normally expressed in human pancreatic cells.^149^ However, it has been identified in association with inflammation caused by *Malassezia folliculitis* .^14^ Dectin-1 functions, particularly in fungal infections, by initiating the Syk-CARD9-NFκB pathway and activating the downstream caspase-8-ASC complex, ultimately leading to the production of IL-1β.^12,150,151^ Alam et al. discovered that this Dectin-1 pathway contributes to the pathogenic pathway of Malassezia through activation of cytokines IL-33 from PDA cells, a downstream target of *Kras*^*G12D*^ in tumor-bearing mice. Synergistically, biochemical factors like ROS can stimulate and amplify IL-33 secretion in collaboration with the mycobiome.^149^ This IL-33 secretion may participate in triggering a type 2 immune response, hastening the pancreatic cancer progression along with reducing survival.^152^

Aykut et al. proposed that *Kras* oncogenic murine models exhibit fungal dysbiosis, exacerbating tumor progression through the activation of the C3 complement cascade via recognition and ligation with mannose on cell walls of *Malassezia* through the mannose-binding lectin. The complement system then participates in the corruption of body immunity, expansion, and apoptosis of tumor cells and therapeutic resistance.^147,148^

In a comparative cohort study examining 16 pancreatic cancer patients who achieved long-term survival (at least 4 years) post-pancreatectomy without recurrence, with eight patients after pancreatectomy and chemical therapy as the control group, we found no significant difference in *Malassezia* abundance in their fecal samples, indicating that *Malassezia* may not influence the prognosis.^153^

Increased *Malassezia spp*. abundance has been observed in IBD, CRC, and HCC patients, suggesting its potential involvement in tumorigenesis. Mechanisms may involve tryptophan metabolism and IL-33-mediated inflammation through the complement cascade activation. However, the clinical importance of *Malassezia* has not been proven yet, awaiting further studies.

### 3.3. Aspergillus

#### 3.3.1. Colorectal cancer

An in silico multi-cohort analysis revealed that *A. rambellii* and other five fungal species were enriched in CRC patients compared with patients with adenoma or normal controls. The enrichment of *A. rambellii* was validated both in vivo and in vitro, with another interesting finding that interactions occur among the enriched microbes. The enriched fungi showed significant co-current features, and the *A. rambellii* also exhibited a correlation with *Fusobacterium nucleatum* and *P. micra* (z score −5.95 and −5.07 respectively).^154^ *A. rambellii* administration promoted tumor growth, weight, and proliferation both in vitro and in a xenograft mice model. They further announced that the panel combing both fungi and bacteria better distinguishes CRC patients from health controls and adenoma patients than pure fungal or bacterial panels.^154^ *A. rambellii* and other two species were among the top 3 fungal species that were significantly enriched in CRC according to Gao et al. Other *Aspergillus* species, that is *A. ochraceoroseus* and *A. flavus*, were also found to have increased in abundance in CRC.^120^

#### 3.3.2. Liver cancer

Aflatoxins have been one of the earliest discovered and studied mycotoxins worldwide, with countless studies reporting their potential pathogenic and carcinogenic effects majorly focusing on two species: *A. flavus* and *A. parasiticus* .^132^ The majority of aflatoxins contributing to liver cirrhosis and HCC are of exogenous origin, particularly in developing countries. Aflatoxins are most commonly absorbed through ingestion and rapidly cleared from blood. AFB1 has been experimentally shown to possess the capability to damage intestinal epithelial cells, leading to the disruption of intestinal structure.^155^ This leads to an influx of immune cells, which may exacerbate tissue damage and impair the absorption of nutrients, particularly vitamins A and E.^156^ Upon absorption, AFB1 is mainly distributed in the liver, while lesser amounts are found disseminating into kidneys and mesenteric vein. Metabolized by the CYP450 system, AFB1 forms DNA adducts, which is its major mechanism for tumorigenesis. AFB1 also induces ROS and inhibits protein, RNA, and DNA synthesis, ultimately leading to apoptosis in microglia via NF-kB activation in oxidative stress.^96^

*A. rambellii*, *A. flavus* and *A. parasiticus* exhibit enrichment in tumors, indicating the potential role of *Aspergillus* in tumor development. AFB1 produced by *A. flavus* and *A. parasiticus* significantly contributes to HCC development by damaging intestinal epithelial cells, impairing nutrient absorption, and inducing oxidative stress, leading to apoptosis and tumorigenesis.

### 3.4. Other fungi

Some fungi species other than those mentioned above are reported to show differences in abundance in cancer patients, despite the fact that they only take up a little account of the gut fungal composition. Chang et al. mentioned that apart from *C. albicans*, *Torulopsis glabrata*, *Torulopsis tomata, C. tropicalis*, *C. krusei*, and *C. parepsilosis*have have also been isolated from esophageal samples.^103^ Considering GI tumors with intratumor lesions, *C. albicans*, *C. tropicalis*, *C. dubliniensis*, and other Candida species could all be distinguished with an increase in abundance, with interactions with certain bacterial and fungal species, such as *Lactobacillus gasseri* and *S. cerevisiae* .^112^

Multiple fungi species, although not directly implicated in tumor progression, are potentially associated with exacerbating diseases that have a high likelihood of progressing into cancer. *Phoma*, another opportunistic pathogen, is found to cause lung mass^36^ and subcutaneous mycosis.^121^ *C. metapsilosis* M2006B demonstrated the ability to alleviate experimental colitis in a large-scale human intestinal fungal cultivation and functional analysis.^117^

## 4. Fungi as biomarkers

Several studies have demonstrated the possibility of utilizing fungi as biomarkers for tumor screening and diagnosis due to the significant difference in fungal composition compared with the healthy control.^146,157^

An important parameter is the *Basidiomycota: Ascomycota* ratio (B/A ratio), a significant increase in B/A ratio is found concerning CRC and ulcerative colitis,^146,158^ although this can be controversial among different studies.^145^ Models incorporating diverse microbiomes have been proposed for predicting diseases like colon adenoma and CRC. They exhibit varying compositions, resulting in differences in accuracy that can be estimated by the area under the receiver-operating characteristic curve (AUROC). However, there are studies showing no difference in fungal composition in CAC.^159^ Decreased diversity of fungi indicating fungal dysbiosis is found in polyp patients, supporting the role of fungi in chronic tumorigenesis process.^121,145^ Luan observed a correlation between malignancy potential of adenoma, and disease stage with changes in fungal composition, highlighting specific fungal species composition at each stage.^121^ Gao et al. reported a non-significant increase in *M. restricta*, with *Leucoagaricus_sp_SymCcos* and *fungal_sp_ARF18* undergoing depletion in adenomas.^120^ In the study conducted by Cocker et al., 14 fungal biomarkers achieved an AUROC of 0.93 that effectively distinguished CRC from controls. However, these fungal markers only achieved AUROCs of 0.63 in classifying adenoma subjects from controls.^146^ Another study discovered that 16 multi-domain markers including 11 bacterial, four fungal, and one archaeal feature achieved good performance in diagnosing patients with early-stage CRC with an AUROC of 0.78.^157^ Gao et al. regarded *Taxomyces andreanae*, *A. rambellii*, *Lachancea_waltii*, *fungal_sp_ARF18*, and *Phanerochaete chrysosporium* as the top five most important biomarkers with the AUROC of 0.926.^154^

Some other cancers were also evaluated for the potential of utilizing gut fungi as tumor biomarkers, including GC and HCC.^111,115,132^

Numerous studies have agreed on the potential that fungal-bacterial interplay influences the tumor development. Cocker et al. announced a significant increase in amount and detrimentality, but also more antagonistic bacterial–fungal interplay in healthy patients compared with CRC patients (*p* < .00001), very similar with the discovery by Liu et al., as well as our previous research, demonstrates that an addition of bacterial biomarkers to the fungal marker can further increase the AUROC of our random forest model in predicting the effect of immune checkpoint inhibitors.^146,157,160^

Two large pan-cancer studies manifested a significant relationship between intra-tumor fungal ecologies and the tumor type and the fungal–fungal interaction or fungal–bacterial interaction as potential indicator of prognosis, such as the increased *Candida-to-Saccharomyces* ratio in late-stage metastatic CRC, and specific fungal–bacterial-immune clusters.^112,161^

## 5. Influence on tumor treatment

### 5.1. Chemotherapy

Researchers have discovered the effect of gut microbiota, especially gram-positive bacteria, in reducing the pathogenic Th17 cell response and rescuing cyclophosphamide resistance.^162,163^ New strategies have been discovered targeting gut microbiota to enhance the efficacy of chemotherapy, including bacterial derivatives and phage-guided nanotechnology.^164,165^

Few studies have been dedicated to the association of the fungal community with the efficiency and resistance of tumor chemotherapy. *C. tropicalis* is associated with CRC and is shown to promote chemoresistance via lactate production and MLH1 inhibition. Inhibiting lactate mitigates the resistance via the GPR81-cAMP-PKA-CREB pathway, regulating MMR protein expression.^139^

### 5.2. Fecal microbiota transplantation

Fecal microbiota transplantation (FMT) is a transformative method that effectively alters recipients’ gut microbiota by transferring a diverse consortium of host-adapted microbial species, encompassing bacteria, bacteriophages, fungi, and their metabolites.^166^ In recent years, FMT has been taken into clinical trials of multiple diseases, both intra-intestinal and extra-­intestinal, including recurrent *C. difficile* infection (CDI), IBD, graft­-versus-­host-­disease (GVHD), irritable bowel syndrome, obesity, and diabetes.^158,167–170^

FMT is able to reverse the reduction in fungi taxa and increase in *C. albicans* in recipients with CDI and ulcerative colitis.^158,167^ Overloading *C. albicans* in donor stool correlates with reduced FMT efficacy in CDI patients.^167^ Meanwhile, recipients with relatively high abundance of gut *C. albicans* respond better to FMT than controls in the condition of ulcerative colitis, and the mechanism may involve the beneficial condition for bacterial diversity recovery.^158^ It is also found to increase the abundance of certain fungi such as *Aspergillus* in CDI^167^ and *Schizosaccharomyces pombe* in ulcerative colitis^171^ after FMT.

The application of FMT has not extended to the treatment of malignancies until now, except for some attempts at improving the efficacy of immunotherapies. Wang et al. presented two case series of immune checkpoint inhibitor-associated colitis successfully treated with FMT, restoring gut microbiota balance, substantially decreasing cytotoxic T cells, accompanying the concomitant increase of regulatory T cells.^172^ Yet, since only two patients were treated and no control groups were involved in the study, more valid evidence should be given to prove its efficiency and safety.^172^

The long-time safety of FMT has not been proven, although studied for years. The known possible adverse events are only limited to mild side effects such as abdominal cramping, bloating, and sore throat, with one death discovered attributed to FMT.^173–175^ More clinical trials and cohorts should be done to explore all the possible adverse effects to ensure its safety.

### 5.3. Fungal- derived products

Bacterial-associated products, including prebiotics, probiotics, and synbiotics, are the most commonly acknowledged microbial adjustment products that may work in tumor prevention and treatment.^176^ Meanwhile, fungal-derived products are also worth mentioning. Citrinin is a mycotoxin derived from polyketides, and it is produced by various fungal strains, including those belonging to the genera *Monascus*, *Aspergillus*, and *Penicillium*. It has been studied for a long time for its potential antineoplastic effect by inducing cell death, reducing DNA repair capacity, and inducing cytotoxic and genotoxic effects.^177,178^ β-glucan administration, whether oral or systemic, has shown promising antineoplastic effects in multiple malignancies including breast cancer, colon cancer, and melanoma, exhibiting benefits in metastasis treatment. The mechanism might involve the induction of tumoricidal peripheral blood monocytes, macrophages, and NK cells, as well as pro-inflammatory cytokines/chemokines including IFN-γ, TNF-a, CXCL9, and CXCL10, and enhanced IRF1 and PD-L1 expression.^179–181^ Marine-derived *Aspergillus* is capable of producing metabolites that are potentially tumoricidal. Asperphenin A inhibits CRC growth by triggering microtubule disassembly and cell cycle arrest, and also has anti-proliferative effect in multiple tumors.^182^ Preussin displayed cytotoxic and anti-proliferative activities in breast cancer cell lines.^183^ β-Galactosidases, known as one of a kind of prebiotics, function as lysosomal exoglycosidases involved in glycoconjugate metabolism. This enzyme can also be isolated from *Aspergillus Terreus* to effectively promote the proliferation of MCF-7 breast cancer cells in vitro.^184^

### 5.4. Radiotherapy

Whether microbiome affects radiotherapy has not been clearly comprehended until now. Commensal microbiomes may differently influence the response to radiotherapy. Depletion of fungi is beneficial for radiotherapy whereas antibiotic-mediated bacterial depletion reduces responsiveness. The immune responses included in these effects are orchestrated by Dectin-1.^185^

### 5.5. Immunotherapy

Immunotherapy is revolutionizing tumor treatment. Strategies have been proposed to augment the potency of drugs and immune generation. Response to PD-1 blockade treatment was observed to have a positive correlation with increasing abundance of *Akkermansia* after FMT in murine models, followed by increased recruitment of CCR9^+^CXCR3^+^CD4^+^ T cells.^186^ FMT also shows positive results in counteracting tumor microenvironment and rescuing anti-PD-1 sensitivity in clinical trials of melanoma.^187^ Β-glucan and its nanoparticles are demonstrated to manipulate the tumor microenvironment, thereby augmenting the response to immunotherapy.^188^ Our recent study also demonstrated the gut bacterial-fungi interplay and the possibility of fungi as biomarker for efficacy of immunotherapy.^160^

Overall, the usage of fungi in the tumor treatment is almost a brand-new field. Recent studies majorly focus on the potential causal relationships of the fungi with the effectiveness, efficacy, and resistance of tumor treatment. Some specific fungi species and fungal products are mentioned, including *C. tropicalis* that promotes chemoresistance in CRC, some fungal-derived products like citrinin and β-glucan exhibiting antineoplastic effects, and *Aspergillus* metabolites that show promise in CRC treatment. But much more about fungi and cancer treatment awaits to be found, especially in the field of FMT and immunotherapy, which are the revolutionizing medical techniques and therapies for disease treatment.

## Conclusion

Although fungi and cancer have been widely studied separately for years, the cancer mycobiome field remains at its starting-line. The techniques and database are far from united and integrated, and there are large amounts of unexplored gut fungi beyond our knowledge. The sample type and size vary greatly between studies and significant amounts of unclassified taxa were mentioned. These limitations have greatly disturbed scientists to find the actual causal relationship between cancer development and fungal changes. Nonetheless, recent studies still help with the discovery of the carcinogenic power of pathogenic fungi, functioning through immune activation, metabolites, toxin production, bacterial–fungal interaction, or biofilm formation. Although the validity, feasibility, and security of the present strategies and investigation methods involving gut mycobiome need to be further addressed, the application of gut fungi to the screening, prevention, and treatment of cancers is a promising field well-worth dedication.

## Data Availability

Data sharing is not applicable to this article as no new data were created or analyzed in this study.
